# Eye, Ear, Nose, and Throat

**Published:** 1895-09

**Authors:** 


### *SELECTIONS AND ABSTRACTS.*

---

### EYE, EAR, NOSE, AND THROAT.

EDITED BY DUNBAR ROY, A.B., M.D.

TRI-CHLORACETIC ACID IN CHRONIC SUPPURATION OF THE EAR. Dr. W. H. Okuneff, St. Petersburg (*Monat f. Ohrenh.*, January, 1895).

Tri-chloracetic acid is superior to others—*e. g.*, chromic acid—in that it does not spread, and after its use syringing with salt solution is unnecessary. The crystalline form of the acid makes its use very difficult, but following Cholewa, I use a common iron wire upon the end of which a loop is formed. A small crystal is engaged in the loop and passed once or twice over the glass of a burning lamp. It requires only a slight warming until the crystal melts in the loop as in a frame. The acid spreads quickly at the point of application, and it is better to remove the superabundant acid by syringing. The cauterization is very painful, so that a $\frac{10}{70}$ or even $\frac{15}{70}$ cocaine solution should be used previously to anesthetize the parts. The previously warmed cocaine solution should be left for two or three minutes in contact with the diseased parts, during which time two or three syringes should be prepared with lukewarm water, and the crystal engaged in the loop of the applicator. Then with the aid of a hard rubber speculum apply the crystal, after drying the ear thoroughly, to the mucous membrane of the middle ear as well as to the edges of the perforation. The mucous membrane of the edges of the perforation become white. Then to prevent the spreading and to remove the superabundant acid, quickly syringe. The patient must meanwhile cleanse the ear at home. After drying the ear I blow aristol, boric, etc., therein. Generally this procedure should be performed one to two times per week. But in those cases where one wishes to promote the growing together of the perforation, cauterization should not be done oftener than every eight or nine days, as too frequent cauterization destroys the new growth of granulation and connective tissue. Between applications syringings with disinfectants are made, or in moderate otorrheas, drops (sol. resorcin 2 per ct.) applied, or the ear is left alone.

I have used this method mainly in cases of chronic suppuration with large perforations in the membrane. In most, treatment by other remedies have failed. Others presented granulations upon the membrane or the adjacent walls of the canal. Finally, in cases where one wished to stimulate the epidermal edges of the perforations, in order to lead to its cicatrization.

In all cases treated as above, the otorrhea, with its usual odor, disappeared. The pus loses quickly its unhealthy color. The granular irregularity disappears, the mucous membrane becoming even and smooth; the small polyps also disappear. But with moderate sized polyps the cauterization fails, and here it is necessary to operate. Dry perforations with epidermal edges can in a shorter time be brought to cicatrization than by the use of other remedies, and more permanently if the middle ear process is at an end. It is not necessary to restore the membrane by cicatrization, but also to restore its qualities of integrity and invulnerability in order to protect the inner parts from external unfavorable influences. Trichloracetic acid has the capability of doing this. Also, after cicatrization the hearing may often, by systematic treatment, be brought on the road to a normal condition. This is especially true in childhood. I believe that in persons from five to twenty-five years of age, by this means, always the end of otorrhea, the cicatrization of the perforations and the return of the function of hearing can be brought about.

In forty-two cases under my personal observation treated by this method, in thirty-eight the otorrhea ceased 90.4 per cent.; in two, marked lessening 5 per cent.; in two, slight lessening 5 per cent.; in twenty-three, cicatrization of the defects and perforations—*i. e.*, in more than half of the cases. As in most of the cases, the cauterizations were not systematically conducted, but were more or less occasional and irregular, so can one suppose that the results would be still more pronounced were the cauterization systematically performed.

Where the physician does not care to bestow a half-hour upon his patient and is not afraid of tediously progressive manipulations, then I repeat, can we always succeed in obtaining the complete cure of the chronic otorrhea and the re-establishment of the hearing.

Very true is this in children, where we can always count upon the regenerative capability of the membrana tympani.—*Annals of Ophthalmology and Otology.*

---

### INTERSTITIAL KERATITIS AND SYNOVITIS WITH A REPORT OF A CASE IN WHICH BOTH WERE UNILATERAL.

G. Crawford Thomson, M.D., Durham (*The Lancet,* April, 1895). The writer reviews the literature of this subject and shows that credit is due to Foerster, who in 1877 "first observed the close clinical connection between the two diseases, and for having first pointed the non-rheumatic character of the joint disease." He then describes in detail a case in which "both affections remained unilateral," a rare exception to the law formulated by Hutchison, that "interstitial keratitis in its typical form is always in the end symmetrical." . . . "As to the causation of both affections, I do not feel justified in attributing them to inherited syphilis simply for the fact that interstitial keratitis existed together with knee-joint affection. The history of my case, complete as it is, contains scarcely anything to suggest syphilis." Complete recovery took place under a simple tonic treatment, neither mercury nor iodide being given. In a disease with a decided tendency to recovery this latter fact is not of much value; still it, in some respects, corroborates the view taken here."

The writer argues against the exclusively specific nature of interstitial keratitis, doubting the correctness of the rule laid down by Hutchison. "Interstitial keratitis in its typical form is always a consequence of syphilis and, in itself, sufficient for the diagnosis." The results of most ophthalmologists, as shown by a recent table of Ogilvie, do not bear out Hutchison's rule. "Another argument against the exclusively specific nature of interstitial keratitis given by Ogilvie, is that it has been observed in dogs and experimentally produced in rabbits. Since the publication of his paper several cases of intertitial keratitis have been observed in bears by Hennicke, and the diagnosis has been confirmed by microscopical examination." "According to Fournier four theories exist concerning the causation of interstitial keratitis: (1) That it is a cachectic malady—une manifestation de la misère organique (Panas); (2) that it is produced by scrofulous or strumous disease (W. McKenzie); (3) that it is exclusively, or nearly exclusively, due to syphilis (Hutchison); and (4) that it is a lesion of natural nutrition (Fournier). He dismisses (2) with little comment, as no connection with strumous disease could ever be proved; he considers (3) to be contrary to clinical facts, as interstitial keratitis is met with apart from any specific influence; and refutes (1) because interstitial keratitis is met with in subjects otherwise apparently healthy, in private as well as in hospital practice. I fail to see that Fournier's explanation differs materially from that of Panas." A lesion of general nutrition, "cachexia," and "organic misery" are only different expressions for the same conditions, or different degrees of the same condition. Therefore, the fact that interstitial keratitis is met with in apparently healthy subjects is explicable by either theory only in one and the same way—viz.: that the defective general condition in these cases is apparent as localized or limited to one special organ. This same view has been recently taken by Panas, who admits that between Fournier's dyscrasia and his cachexia practically no difference exists, and that he therefore willingly accepts Fournier's denomination instead of his. From the above the conclusion has to be drawn that in the production of interstitial keratitis, hereditary syphilis is probably operative in between 60 and 70 per cent.

I therefore from my own case, as well as from the experience of all and Lavergne, draw the following conclusions: Neither interstitial keratitis nor the synovitis, nor their coexistence, is itself absolute proof of hereditary syphilis; that the joint disease also is due to a general defect of nutrition, in the production of which inherited syphilis plays a prominent part, the extent of which we are not able to give in figures. In what this disturbance of general nutrition consists, what general condition constitutes the connecting link between interstitial keratitis and synovitis, is merely a matter of conjecture.—*Annals of Ophthalmology and Otology.*

### RECENT EXPERIENCES IN THE TREATMENT OF DETACHED RETINA.

C. S. Bull, of New York, draws the following conclusions from his own experience:

1. The science and practice of ophthalmology have as yet discovered no better means for dealing with detachment of the retina than the old methods which have been advised and carried out for so many years—viz: rest on the back in bed, atropine, a bandage, and the internal administration of some drug which may induce absorption of the subretinal fluid.

2. The continual use of pilocarpine, either hypodermically or by the mouth, may cause great prostration, even in cases where it is apparently well borne; and the desired effect may sometimes be produced by small does of sodium bicarbonate or potassium iodide largely diluted with water.

3. In all recent cases, puncture of sclera subconjunctivally may do good temporarily, by letting out the subretinal fluid and allowing the retina to collapse, thus producing some improvement in the vision. But the apparent improvement is generally transient, and when membranes exist in the vitreous no improvement can be expected from simple puncture.

4. Division of the fixed membranous opacities in the vitreous causes but little reaction and may do positive good, even without division of the detached retina, as it reduces the danger of extension of the detachment. It is positively contraindicated in cases where the vitreous opacity is vascularized, as it would certainly induce free hemorrhage into the vitreous. It should never be done when there is an irritated or inflamed condition.

5. Division of the detached retina may always be done in a quiet eye and causes little or no reaction. If membranous bands are present in the vitreous, these should always be divided at the same time.

6. In most cases all of these operative procedures produce but temporary improvement, and in many cases no effect whatever is gained by them.

7. There seems no good reason for further indorsement of the method advocated by Schöler, but every reason for rejecting it from the domain of ophthalmic surgery.

